# Solving the Riddle of Sudden Cardiac Death in Hypertrophic Cardiomyopathy: The Added Role of Cardiac Magnetic Resonance

**DOI:** 10.3390/jcdd10060226

**Published:** 2023-05-23

**Authors:** Kamil Stankowski, Stefano Figliozzi, Costanza Lisi, Federica Catapano, Cristina Panico, Francesco Cannata, Riccardo Mantovani, Antonio Frontera, Renato Maria Bragato, Giulio Stefanini, Lorenzo Monti, Gianluigi Condorelli, Marco Francone

**Affiliations:** 1 Department of Biomedical Sciences, Humanitas University, Via Rita Levi Montalcini, 4, Pieve Emanuele, 20090 Milano, Italy; 2 Humanitas Research Hospital IRCCS, Via Alessandro Manzoni, 56, Rozzano, 20089 Milano, Italy

**Keywords:** hypertrophic cardiomyopathy (HCM), sudden cardiac death (SCD), cardiac magnetic resonance (CMR), right ventricle, mapping, late gadolinium enhancement (LGE), implantable cardioverter defibrillator (ICD), multimodality imaging

## Abstract

Cardiac magnetic resonance (CMR) has been recently implemented in clinical practice to refine the daunting task of establishing the risk of sudden cardiac death (SCD) in patients with hypertrophic cardiomyopathy (HCM). We present an exemplificative case highlighting the practical clinical utility of this imaging modality in a 24-year-old man newly diagnosed with an apical HCM. CMR was essential in unmasking a high risk of SCD, which appeared low-intermediate after traditional risk assessment. A discussion examines the essential role of CMR in guiding the patient’s therapy and underlines the added value of CMR, including novel and potential CMR parameters, compared to traditional imaging assessment for SCD risk stratification.

## 1. Introduction

Hypertrophic cardiomyopathy (HCM) is the most common inherited heart disease (prevalence 1:200–1:500) and is characterized by an increased left ventricular (LV) wall thickness in the absence of abnormal loading conditions [1,2]. A considerable variety affects genetic background, morphological expressions, clinical manifestations, and severity of disease progression. Despite a generally benign prognosis [3], some HCM patients are at risk of heart failure and/or sudden cardiac death (SCD). Risk stratification for SCD is paramount to guide patients’ selection for an implantable cardioverter defibrillator (ICD). Risk scores based on conventional clinical and imaging variables [4] have shown suboptimal accuracy in predicting SCD [5,6]. Novel imaging parameters, together with genetic and biochemical signatures, may be instrumental in implementing risk stratification for relevant clinical outcomes [7]. In particular, the introduction of cardiac magnetic resonance (CMR) allows the unveiling of myocardial fibrosis through late gadolinium enhancement (LGE), whose presence and extent have been associated with malignant ventricular arrhythmias [8,9], on top of the accurate measurement of imaging parameters also detectable by echocardiography. The present paradigmatic case report and the following literature review focus on the added role of CMR in SCD risk stratification in HCM patients.

## 2. Case Report

**Figure 1:**
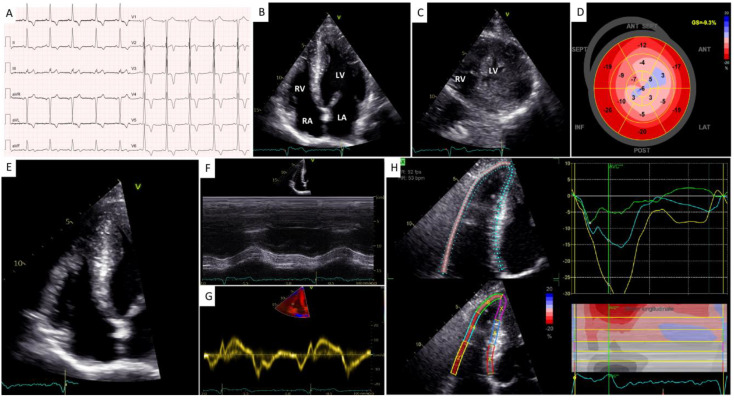
Initial work-up with electrocardiography and transthoracic echocardiography. (**A**) A 12−lead electrocardiogram. (**B**) A 2−dimensional (2D) transthoracic echocardiography (TTE) 4−chamber apical view showing biventricular hypertrophy with an “ace of spades” morphology of the left ventricular (LV) cavity. (**C**) A 2D−TTE parasternal short-axis view at the level of the apex showing biventricular hypertrophy. (**D**) A 2D speckle-tracking echocardiography (STE) map showing reduced global longitudinal strain in the mid-apical segments of the left ventricle. (**E**) A 2D right ventricle (RV) −focused 4−chamber apical view. (**F**,**G**) Conventional indices of longitudinal RV systolic function, i.e., TAPSE (**F**) and S’ TDI (**G**). (**H**) A 2−D STE map showing reduced RV free wall longitudinal strain.

A 24-year-old man underwent a cardiological visit because of exertional dyspnea. He rarely engaged in sports, but for four months, he had noted sporadic shortness of breath during ordinary efforts with mild limitation of his physical activity in the absence of chest pain, palpitations, or syncope. His past medical history was non-significant. The patient denied cardiovascular risk factors or a family history of SCD or cardiomyopathy. His blood pressure was 120/80 mmHg, and his heart rate was 70 bpm. His physical examination was within normal limits. A 12-lead ECG showed sinus rhythm, normal atrioventricular conduction, LV hypertrophy, and inverted T waves in all leads but aVR, D3, and V1 (Figure 1A).

Trans-thoracic echocardiography (TTE) showed an “ace of spades” LV morphology with apical hypertrophy (maximum LV wall thickness equal to 25 mm, Figure 1B,C) associated with mid-ventricular obstruction (peak gradient 38 mmHg). Left ventricular ejection fraction (LVEF) was preserved (62%) while LV global longitudinal strain (GLS) was significantly impaired (−9.3% with a “reverse apical sparing” pattern, Figure 1D). There was no systolic anterior motion of the anterior mitral valve leaflet nor LV outflow tract obstruction at rest or during Valsalva. Second-degree diastolic dysfunction and left atrial enlargement (maximum volume: 42 mL/m^2^) were observed. There was also right ventricular (RV) hypertrophy, with a maximum RV wall thickness equal to 8 mm. The conventional indices of RV systolic function were normal (i.e., TAPSE 26 mm, S’ TDI 12 cm/s, FAC 49%, Figure 1E–G). In contrast, RV free wall longitudinal strain (RVFWLS) was significantly impaired (–16%, Figure 1H).

**Figure 2:**
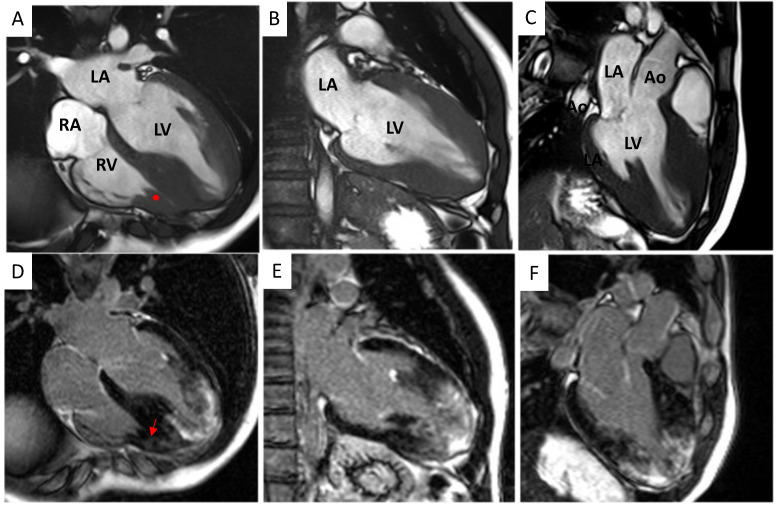
Cardiac magnetic resonance morphological and fibrosis characterization. (**A**–**C**) Balanced Steady State Free Precession (bSSFP) cine 4−, 2− and 3−chamber views showing hypertrophy of the mid-apical segments of the left ventricle. Hypertrophy of the right ventricular wall and a right ventricular papillary muscle (red asterisk; panel (**A**)) is also noted. (**D**–**F**) Magnitude reconstruction (MAG) LGE 4−, 2− and 3−chamber views showing extensive late gadolinium enhancement (LGE) of the left ventricular mid-apical segments and the right (red arrow; panel (**D**)) ventricle. Ao = aorta, LV = left ventricle, RV = right ventricle, LA = left atrium, and RA = right atrium.

**Figure 3:**
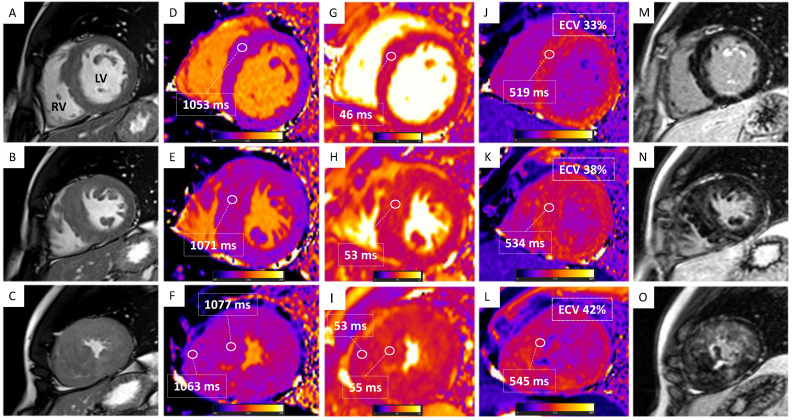
Multiparametric cardiac magnetic resonance tissue characterization. Basal (upward), mid (mid), and apical (downward) short-axis views are shown. (**A**–**C**) Balanced Steady State Free Precession (bSSFP) short-axis views showing hypertrophy of the mid-apical segments of the left ventricle (LV) and hypertrophy of the right ventricular (RV) wall. (**D**–**F**) Modified Look-Locker inversion recovery (MOLLI) T1-mapping showing elevated native T1 with increasing values from base to apex; elevated T1 values of the RV free wall are also shown. (**G**–**I**) True fast imaging with steady-state precession (True-FISP T2) mapping showing elevated native T2 values at the apex. (**J**–**L**) MOLLI post-contrast T1-mapping showing elevated post-contrast T1 and elevated extracellular volume (ECV) with increasing values from base to apex. (**M**–**O**) Magnitude reconstruction (MAG) late gadolinium enhancement (LGE) showing LGE of the mid-apical segments of the LV and RV.

A 1.5 Tesla CMR system confirmed bi-ventricular hypertrophy (maximum LV and RV wall thickness equal to 27 mm at the septal segment of the apex and 12 mm, respectively) with preserved pump function (LVEF 64%; right ventricular ejection fraction (RVEF) 66%, Figure 2 and Figure 3A–C). The hypertrophy of an RV papillary muscle was also evident. At tissue characterization, elevated native T1 (normal values below 1014 ms) and T2 (normal values below 52 ms) values were noted both in the apical LV segments (1077 +/− 45 ms and 55 +/− 6 ms, respectively) and RV segments (1063 +/− 51 ms and 53 +/− 3 ms, respectively, Figure 3D–I). Increased global extracellular volume (ECV, normal values below 30%) was also noted (ECV = 42%, Figure 3J–L). There was a significant amount of patchy LGE mostly in the apical segments of LV (28% of LV mass) and RV (Figure 2D–F and Figure 3M–O). All alterations of tissue parameters exhibited a base-apex gradient; Figure 2 and Figure 3. At feature-tracking analysis, there was a reduction of peak global longitudinal strain (−7.4%), circumferential strain (−12.1%), and radial strain (19.7%).

The 48 h Holter EKG monitoring showed normal sinus rhythm, no sustained or non-sustained ventricular arrhythmias, with a premature ventricular complex burden <5%. Laboratory examinations revealed mildly increased high-sensitivity troponin I (40 ng/L, normal value < 19.8 ng/L) and BNP (155 pg/mL, normal value < 100 pg/mL).

Genetic testing showed a class IV variant (likely pathogenetic) in the MYBPC3 gene (c.482dupC), causing a premature stop codon to form.

After a multidisciplinary meeting, the patient underwent a subcutaneous ICD (s-ICD) implantation for primary prevention. He was also advised against participation in competitive sports and discouraged from intense physical activity. The patient was started on bisoprolol, up to 3.75 mg daily, with improvement of his dyspnea (NYHA I-II) at the 9-month follow-up, with no arrhythmic events at device interrogation. The beta-blocker was not further up-titrated due to bradycardia.

## 3. Case Discussion

Identifying the subgroup of HCM patients at risk of SCD is daunting for physicians in daily practice [10]. Traditional risk stratification algorithms [4] based on conventional clinical risk factors (i.e., age, unexplained syncope, family history of SCD, and non-sustained ventricular tachycardia) and simple imaging parameters (i.e., LA size, maximum LV wall thickness, and maximum LVOT gradient) might lack sensibility and accuracy. Accordingly, an ESC HCM SCD risk ≥6% showed a sensitivity of 32% (95% CI 19.1–47.1%) in a retrospective study including 784 HCM adult patients. In the same population, the AHA/ACC algorithm, which encompasses novel risk factors, showed better sensitivity (96%; 95% CI 85.5–99.5%) at the price of a reduced specificity (59%; 95% CI 55–62.2% vs. 95%; 95% CI 93.1–96.4%) compared to the ESC HCM SCD risk score [6].

The present case is emblematic and exemplificative in underlying the added role of CMR in SCD risk stratification in patients with HCM, especially in those at low-intermediate risk for SCD on traditional assessment. Such HCM patients, including the one reported in the present manuscript, would not be captured at significant risk of SCD and would remain unprotected from SCD based only on echocardiographic imaging assessment. In these cases, the addition of CMR allows the physicians to refine SCD risk, leading to a dramatic impact on clinical decision-making (i.e., ICD implant), thanks to the high accuracy of CMR in measuring imaging parameters that are also detectable at echocardiography, together with the unique capability of the method to unveil the burden of myocardial fibrosis, which is growlingly recognized an essential risk factor for malignant ventricular arrhythmias.

Thus, CMR has recently been upgraded for SCD risk stratification in the latest international guidelines. This examination is initially indicated for patients with HCM who are not otherwise identified as high risk for SCD or whose ICD implant remains uncertain after conventional clinical and echocardiographic assessment. Follow-up CMR (every three to five years) is also indicated for non-high-risk HCM patients to update SCD risk stratification in parallel with potential changes regarding LGE, LVEF, maximum left ventricular wall thickness (MLVWT), and the development of apical aneurysms [11].

**Figure 4:**
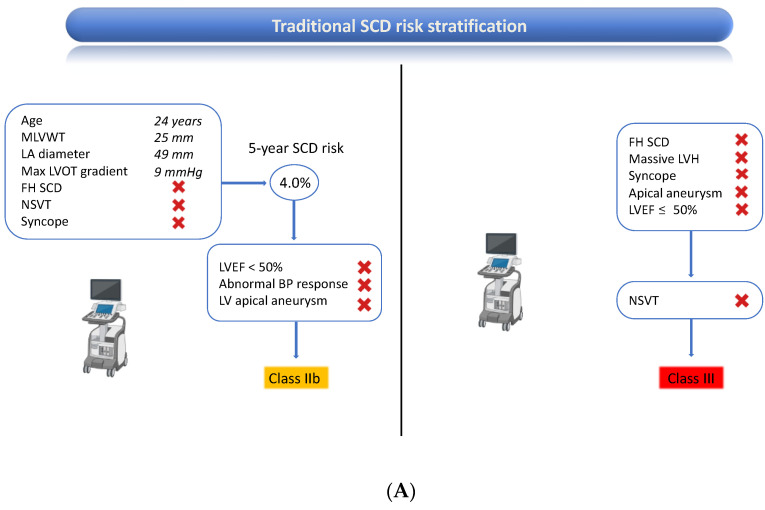

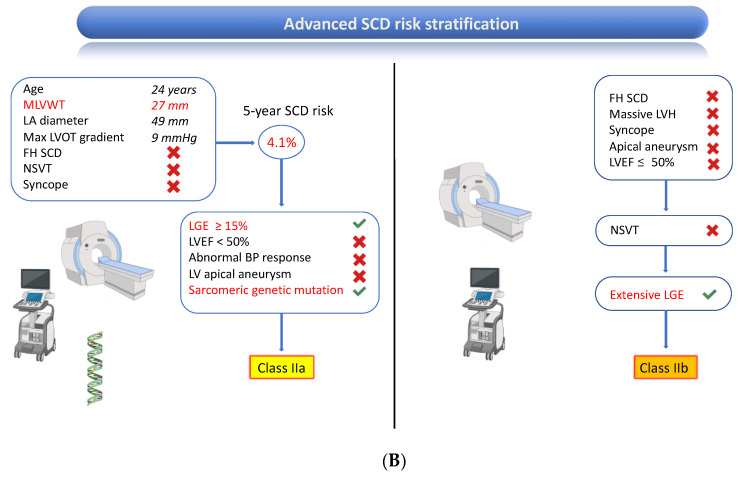
Sudden cardiac death risk stratification and class of recommendation to ICD in the present patient according to traditional assessment (panel (**A**)) and advanced assessment including CMR (panel B). For both panels, the European Society of Cardiology and American Heart Association/American College of Cardiology algorithms are respectively shown on the left and right side of the figure. Parameters that changed between baseline and advanced evaluation are colored in “red”(panel (**B**)). BP: blood pressure; FH: family history; ICD: implantable cardiac defibrillator; LA: left atrium; LGE: late gadolinium enhancement; LV; left ventricle; LVEF: left ventricular ejection fraction; LVH: left ventricular hypertrophy; LVOT: left ventricular outflow tract; MLVWT: maximum left ventricular wall thickness; NSVT: non-sustained ventricular tachycardia; SCD: sudden cardiac death.

Based on traditional risk assessment, the ESC HCM SCD risk score [4] entailed a five-year risk of SCD ≥ 4% but <6% in the present patient, which constitutes a class IIb indication for ICD [12]. The latest European guidelines also mandate evaluating additional risk factors, not included in the risk score, such as the presence of sarcomeric pathogenetic genetic mutations or extensive LGE, defined as ≥15% of LV mass, which were present and allowed re-classifying the indication to class IIa. According to the AHA/ACC HCM guidelines [11], the patient lacked all five major conventional risk factors for SCD. However, CMR intercepted extensive LGE, which indicated ICD implantation; Figure 4.

Established and promising imaging parameters useful for SCD risk stratification are reported in Table 1.

The European and American guidelines contemplate the following parameters detectable by CMR for SCD risk stratification in HCM patients: LVEF, MLVWT, LA size (expressed as anteroposterior diameter), the presence of an apical aneurysm and LGE (expressed as a percentage of LV mass) [11,12]. For the assessment of several of these imaging parameters, CMR shows unique strengths as compared to echocardiography.

### 3.1. Established Imaging Markers of an Increased Risk of SCD

#### 3.1.1. Maximum Left Ventricular Wall Thickness

CMR is more accurate and sensitive than echocardiography in assessing LV wall thickness. Accordingly, CMR can unmask hypertrophic segments that are missed by echocardiography. Maron et al. [13] found that in 12% of HCM patients, CMR was able to identify hypertrophied segments that were not detected by echocardiography. The segments where CMR outperformed echocardiography were the anterolateral wall, the posterior portion of the ventricular septum, and the LV apex.

Conversely, the reduced accuracy of echocardiography compared to CMR might also result in an overestimation of LV wall thickness. In a cohort of 195 HCM patients [14], the overestimation of LV wall thickness by echocardiography was secondary to the inclusion of RV muscular structures (60% of cases) or, less commonly, to the inclusion of LV trabeculations, papillary muscle, apical-septal bundle, or imaging plane obliquity. The disagreement between echocardiography and CMR in the measurement of MLVWT was higher in apical HCM patients, and in 16% of the patients, the discrepancies occurred at diagnostic (15 mm) or prognostic (30 mm) thresholds, significantly affecting clinical management.

MLVWT as a prognostic marker is subject to considerable intra- and inter-reader variability, even when image quality and operator experience is high, resulting in inappropriate decisions concerning ICD implantation potentially being made in one in seven patients, as demonstrated by Captur et al. [15]. Comparing CMR to echocardiography, though, the inter-reader variability by CMR is less than that of echocardiography (1-SD percentage variability of ± 11% vs. ± 20%, respectively). Implementation of artificial intelligence might improve measurement standardization, as demonstrated in a study where the accuracy of machine learning measurement of MLVWT was superior to that performed by human experts [16].

#### 3.1.2. Late Gadolinium Enhancement

A unique advantage of CMR compared to echocardiography is the capability to detect myocardial fibrosis through LGE, which has been associated with an increased risk of SCD in several non-ischemic cardiomyopathies [17,18,19,20,21,22,23,24,25,26].

More than half of HCM patients exhibit LGE, most commonly involving hypertrophied segments of the LV [8,10], and LGE can be quantified as a percentage of LV mass [27].

A seminal study by Chan et al. [8] found a continuum between LGE by percent of LV mass and SCD event risk in HCM patients (adjusted HR 1.46 per 10% increase in LGE, 95% CI 1.12–1.92; *p* = 0.002) and LGE ≥ 15% of LV mass demonstrated a two-fold increase in SCD event risk in those patients otherwise considered at lower risk, with a rate of SCD of 6% at five years. On the other hand, the absence of LGE reduced the risk of SCD (adjusted HR 0.39; 95% CI 0.18–0.84; *p* = 0.02). Especially in low-mid risk patients, as per conventional criteria, CMR can correctly re-classify their future risk of potentially lethal ventricular arrhythmias and allow timely implantation of an ICD in primary prevention. The prognostic value of LGE in HCM patients has been confirmed in a meta-analysis including almost 3000 patients, where both the presence and extent of LGE were associated with SCD and all-cause mortality, among others, independently of baseline characteristics; in particular, the adjusted HR was 1.36 per 10% of LGE (95% CI 1.10–1.69; *p* = 0.005) [9].

A study by Weissler-Snir et al. [28] found that none of the conventional risk factors nor the ESC risk score were predictive of appropriate ICD therapy during a median follow-up of 6.1 years. However, in a subgroup of patients undergoing CMR, it was found that nearly 20% of patients with LGE ≥ 15% of LV mass received appropriate ICD therapies with a five-year cumulative probability of 26.6% compared with only 3.7% in patients with LGE < 15% of LV mass. Strikingly, LGE was the variable with the highest prognostic value for adverse outcomes in a recent network meta-analysis [7], including 58732 HCM patients, resulting as superior to all other associates except New York Heart Association functional class > II.

Conventionally, an LGE cutoff ≥15% of LV mass is considered a risk factor for SCD [11,12]. However, the findings of a recent investigation on 203 HCM patients, assessed by CMR and evaluated with a medium follow-up of more than ten years, suggest that a significantly increased risk of SCD is present for patients showing LGE in more than 5% of LV mass. Those patients showed SCD rates of 5.5% at five years, 13.0% at ten years, and 33.3% at 15 years. Conversely, patients with no or ≤5% LGE of LV mass had a good prognosis [29]. Notably, myocardial fibrosis is a progressive process as LGE extent tends to increase over time, especially in patients with more severe disease at the baseline, and the extent of LGE progression significantly correlates with ICD implantation, LVEF reduction below 50%, and heart failure admission; thus serial CMRs could prompt appropriate management decisions [30].

Although the process of LGE quantification has been subject to methodological discrepancies among different centers, semi-automated algorithms, including myocardial regions with a signal intensity five standard deviations higher than the remote myocardium after contrast injection, are commonly used in everyday practice [31].

Evaluation of the pattern of LGE at CMR is also key in the diagnostic process, especially in the differential diagnosis of other cardiomyopathies where hypertrophy is present such as cardiac amyloidosis, where global subendocardial or transmural LGE is frequently observed [32], and Fabry disease, where the typical pattern is mid-myocardial LGE in the basal to mid infero-lateral wall [33].

#### 3.1.3. Apical Aneurysm

CMR has shown superior sensitivity in detecting LV apical aneurysms/apical thinning compared to echocardiography, whose sensibility is less than 70%, especially for small aneurysms (defined as <20 mm) [34]; however, recurrence to contrast echocardiography increases the sensitivity of the method [35].

#### 3.1.4. Left Ventricular Ejection Fraction

The asymmetric hypertrophy of HCM patients renders Simpson’s biplane method of discs inaccurate, given that this echocardiographic approach makes incorrect geometric assumptions about regular and symmetric LV orientation and cross-sectional shape. Accordingly, CMR has shown superior reproducibility compared to echocardiography when assessing LV dimensions and function. Grothues et al. [36], in a cohort of 60 subjects undergoing duplicate CMR and echocardiographic studies, 20 of whom with LV hypertrophy, showed excellent inter-study reproducibility of CMR measurements of LV volumes and mass as well as superior reproducibility of CMR measurements compared to 2D echocardiographic parameters. CMR, when available, is the most suitable imaging modality to promptly detect a change in LV volumes and, consequently, LVEF during the follow-up of HCM patients.

#### 3.1.5. Left Atrial Size

Left atrium size, expressed as anteroposterior diameter, is considered in the ESC HCM risk score. As of today, LA volume is the preferred method to quantify LA sizes, given that LA diameter is not a reliable size marker in patients with asymmetrical enlargement of the LA [37]. CMR is considered the gold standard for LA volume quantifications through the summation of the disks method [38]. The possibility to orientate focused long-axis imaging on the major axis of the LA allows for obtaining more accurate volumes than with standard long-axis views, with less time employed than with the gold standard summation of disks method [39]. Echocardiography systematically underestimates LA volumes when compared to CMR [40,41]. However, no study has systematically evaluated the prognostic impact of different methods used to assess LA sizes, including the CMR gold standard. This gap in knowledge precludes a systematic evaluation of LA sizes through dedicated LA imaging protocols in current daily practice.

### 3.2. Promising Imaging Markers to Improve the Risk of SCD

#### 3.2.1. T1-Mapping, T2 mapping, and Extracellular Volume

Although LGE evaluation is the reference standard for in vivo assessment of myocardial scar, depicting the relative difference between enhancing areas of fibrosis and normal myocardium, diffuse fibrosis can go undetected on LGE maps because of the absence of normal reference myocardium and because identification of microscopic interstitial fibrosis is limited by the spatial resolution of LGE images [42]. Quantitative assessment of native T1 relaxation time maps, on the other hand, can detect diffuse myocardial alterations: in particular, in HCM, elevated native T1 values correspond to increased interstitial space due to diffuse fibrosis beyond those detected by LGE.

A recent longitudinal study including 203 HCM patients found that, at multivariate analysis, native T1 by CMR was associated with a combined clinical endpoint including cardiac death, transplantation, heart failure admission, and ICD implantation (HR 1.446; 95% CI 1.195–1.749; *p* < 0.001), maintaining the association (HR 1.532; 95% CI 1.221–1.922; *p* < 0.001) in a subgroup of patients judged low risk as per European and American guidelines [43].

Extracellular volume (ECV) fraction, derived from the myocardium and blood T1 values before and after gadolinium administration as well as the patient’s hematocrit, reflects the excess collagen deposition in the extracellular space. A recent study with a small sample size demonstrated that ECV was the best CMR parameter to identify patients with HCM-SCD risk ≥ 4% [44]. Another recent study with limited sample size and longitudinal follow-up has shown that global ECV was an independent predictor of a composite outcome, including SCD, heart transplant, cardiopulmonary resuscitation after syncope, unplanned readmission for heart failure, and abnormal discharges according to programmed control after ICD implantation at multivariate analysis (HR, 1.27 [95% CI: 1.10–1.47]) [45]. Prolonged values at T2 mapping, potentially signaling fibrosis or edema due to ischemia or microvascular dysfunction, can be found in HCM patients [46]. It is accepted that tissue remodeling may precede morphological alterations; however, data on the prognostic significance of T2 are lacking.

#### 3.2.2. Right Ventricular Involvement

RV hypertrophy can be identified in 31–44% [47,48] of HCM patients, and its mechanism is either a biventricular effect of HCM-associated mutations or postcapillary pulmonary hypertension. Notably, CMR is considered the gold standard imaging modality for morpho-functional RV assessment [49]. A recent investigation including 290 HCM patients with preserved LV ejection fraction found reduced RVEF [HR 1.10 (95% CI 1.06–1.15) and RV longitudinal strain [HR 1.05 (95% CI 1.01–1.09) at CMR as independent predictors of non-sustained ventricular tachycardia [50]. In another study, RV hypertrophy by CMR was significantly and independently associated with a composite clinical endpoint, including admission for heart failure, ventricular tachyarrhythmia/fibrillation, stroke, and SCD (HR 8.7; 95% CI 2.7–28.1) [51]. RVEF, RV strain, but not RV volumes, have also been associated with a combined clinical endpoint, including heart failure-related hospitalizations, death, or aborted SCD [52].

RV-LGE might be challenging to assess, given the thin-walled structure of the RV. As of today, there is no link between RV-LGE and SCD in HCM patients. Overall, the clinical implications of RV involvement by CMR on the risk of SCD remain undefined.

#### 3.2.3. Left Ventricular Strain

Feature tracking CMR is a promising tool for detecting systolic functional abnormalities occurring earlier than LVEF impairment [53]. In a recent investigation including 293 HCM patients with a median follow-up of 15.0 months, 14 experienced a composite outcome, including eight all-cause deaths, four resuscitated cardiac arrests, and two cardiac transplantations. Peak systolic global longitudinal strain rate by CMR resulted in being independently associated with clinical outcomes (HR 15.297, *p* < 0.001) after adjusting for conventional clinical characteristics, echocardiography, and CMR parameters, showing incremental prognostic value over conventional variables [54]. In another recent article, a global radial peak strain value of <27% showed the best area under the ROC curve and remained independently associated with ventricular tachycardia after adjustment for confounders (OR 7.33, 95% CI 1.07 to 50.41, *p* = 0.043) [55]. Larger sample size studies and focused study endpoints remain necessary to define the potential value of feature tracking CMR in SCD risk stratification in HCM patients.

#### 3.2.4. Left Ventricular Mass

As mentioned above, LV hypertrophy has been mainly evaluated by measuring MLVWT. However, hypertrophy can be asymmetrically distributed in HCM patients, and genotype–phenotype correlations suggest that a wide spectrum of wall thickness can be found in patients with HCM-related gene mutations [13]. LV mass may be an alternative and even more robust marker of LVH than MLVWT, better reflecting the total burden of hypertrophy. CMR is the gold standard technique for LV mass quantification due to its high accuracy and reproducibility [56,57]. Accordingly, Olivotto et al. demonstrated that an increased LV mass indexed for body surface at CMR was more sensitive (100%, with 39% specificity), compared to MLVWT > 30 mm (90%, with 41% sensitivity), in predicting HCM-related death in 264 patients with a follow-up of 2.6 ± 0.7 years. Among the ten patients who died, five of them experienced SCD. Notably, LV mass correlated weakly with MLVWT (r^2^ = 0.38; *p* < 0.001) [58]. Thus, LV mass indexed might be complementary to MLVWT to improve SCD stratification.

#### 3.2.5. Left Atrial Function

In addition to left atrial dimensions, which are already part of the current risk prediction algorithms, impaired left atrial strain, or components thereof (reservoir, conduit, or booster strain), are considered emerging early markers of diastolic dysfunction, which have been associated with several clinical outcomes, but not with SCD so far [59,60].

## 4. Conclusions

Prediction and prevention of SCD in HCM is a major challenge for physicians in daily practice. Despite still being limited by issues in terms of costs and availability, CMR has emerged over the last two decades as a powerful tool uniquely suited for this purpose, given the high accuracy in measuring traditional imaging parameters beyond the unique capability of providing non-invasive tissue characterization. Recent international guidelines have upgraded the value of CMR in SCD risk stratification algorithms. An emblematic case report highlights the added role of CMR in SCD risk stratification in HCM patients, showing its crucial contribution in unmasking high-risk patients who would not be otherwise identified on traditional clinical and imaging assessment.

## Figures and Tables

**Table 1:** CMR value for sudden cardiac death risk stratification in patients with hypertrophic cardiomyopathy.

Imaging Parameter	Clinical Evidence Supporting Assessment for SCD Risk Stratification	CMR Advantages over Echocardiography
MLVWT *	++++	+++
LGE (extent) *	++++	++++
LVEF *	+++	+++
LV aneurysm *	+++	++
Maximum LVOT Gradient *	+++	-
LA size *	+++	++
LV strain	++	+/−
LV mass	+	++++
Extracellular Volume	+	++++
Native T1 mapping	+/−	++++
Native T2 mapping	-	++++
Right Ventricular Involvement	+/−	+++
LA function	-	+/−

- Absent; +/− Doubtful; + Weak; ++ Moderate; +++ Strong; ++++ Very Strong. CMR: cardiac magnetic resonance; LA: left atrium; LGE: late gadolinium enhancement; LV: left ventricle; LVEF: left ventricular ejection fraction; LVOT: left ventricular outflow tract; MLVWT: maximum left ventricular wall thickness; SCD: sudden cardiac death. * Included in international guidelines for clinical management of patients with hypertrophic cardiomyopathy.

## Data Availability

Not applicable.
